# A repeated triple lysine motif anchors complexes containing bone sialoprotein and the type XI collagen A1 chain involved in bone mineralization

**DOI:** 10.1016/j.jbc.2021.100436

**Published:** 2021-02-19

**Authors:** Jeff P. Gorski, Nichole T. Franz, Daniel Pernoud, Andrew Keightley, David R. Eyre, Julia Thom Oxford

**Affiliations:** 1 Center of Excellence in Mineralized Tissue Research, School of Dentistry, University of Missouri-Kansas City, Kansas City, Missouri, USA; 2 Department of Oral and Craniofacial Sciences, School of Dentistry, University of Missouri-Kansas City, Kansas City, Missouri, USA; 3 Department of Ophthalmology and Proteomics Core Facility, University of Missouri-Kansas City School of Medicine, Kansas City, Missouri, USA; 4 Department of Orthopaedics and Sports Medicine, University of Washington, Seattle, Washington, USA; 5 Department of Biological Sciences, Center of Biomedical Research Excellence in Matrix Biology, Boise State University, Boise, Idaho, USA

**Keywords:** N-terminal domain, type XI collagen, bone sialoprotein, alternative splicing, collagen, extracellular matrix, confocal microscopy, cell culture, bone, peptides, AEBSF, 4-(2-aminoethyl)benzenesulfonyl fluoride hydrochloride, BGP, β-glycerolphosphate, BMF, biomineralization foci, BSP, bone sialoprotein, CHAPS, (3-((3-cholamidopropyl) dimethylammonio)-1-propanesulfonate), Column Buffer, 0.05 M sodium acetate buffer (pH 5.2) containing 8 M urea and 0.02% sodium azide, FAM, 6-carboxyfluorescein, Npp, N-propeptide, NTD, N-terminal domain, PVDF, polyvinylfluoride, TBST, Tris-buffered saline containing Tween-20, VR, variable region

## Abstract

While details remain unclear, initiation of woven bone mineralization is believed to be mediated by collagen and potentially nucleated by bone sialoprotein (BSP). Interestingly, our recent publication showed that BSP and type XI collagen form complexes in mineralizing osteoblastic cultures. To learn more, we examined the protein composition of extracellular sites of *de novo* hydroxyapatite deposition which were enriched in BSP and Col11a1 containing an alternatively spliced “6b” exonal sequence. An alternate splice variant “6a” sequence was not similarly co-localized. BSP and Col11a1 co-purify upon ion-exchange chromatography or immunoprecipitation. Binding of the Col11a1 “6b” exonal sequence to bone sialoprotein was demonstrated with overlapping peptides. Peptide 3, containing three unique lysine-triplet sequences, displayed the greatest binding to osteoblastic cultures; peptides containing fewer lysine triplet motifs or derived from the “6a” exon yielded dramatically lower binding. Similar results were obtained with 6-carboxyfluorescein (FAM)-conjugated peptides and western blots containing extracts from osteoblastic cultures. Mass spectroscopic mapping demonstrated that FAM-peptide 3 bound to 90 kDa BSP and its 18 to 60 kDa fragments, as well as to 110 kDa nucleolin. In osteoblastic cultures, FAM-peptide 3 localized to biomineralization foci (site of BSP) and to nucleoli (site of nucleolin). In bone sections, biotin-labeled peptide 3 bound to sites of new bone formation which were co-labeled with anti-BSP antibodies. These results establish the fluorescent peptide 3 conjugate as the first nonantibody-based method to identify BSP on western blots and in/on cells. Further examination of the “6b” splice variant interactions will likely reveal new insights into bone mineralization during development.

Bone is a unique mineralized tissue which supports the body’s organs and muscles yet remains vital throughout an organism’s lifetime. Osteoblasts synthesize osteoid, the extracellular collagenous matrix of bone and direct the deposition of hydroxyapatite crystals within it during bone formation. Type I collagen is the main organic component of bone osteoid. However, it is also enriched with phosphoproteins, glycosaminoglycans, and presumptive nucleators of mineralization (1, 2, 3). It has long been hypothesized that certain structural interactions are required to induce bone mineralization *in vivo*. Two basic types of bone are known, *e.g.*, cortical and trabecular. Each is believed to be formed by different mechanisms referred to as collagen-mediated mineralization (cortical bone) and vesicle-mediated mineralization (trabecular) (4) although some of the same phosphoprotein nucleators have been suggested to play a role in both mechanisms. Despite many efforts, the mechanism of bone mineralization is still unresolved.

With respect to collagen-mediated mineralization, Glimcher and Veis hypothesized that a phosphoprotein nucleator bound near the gap region of fibrillar type I collagen mediates initial nucleation and deposition of mineral crystals (5, 6, 7, 8). While the identity of this hypothetical phosphoprotein has never been identified, bone sialoprotein represents a candidate based on a large body of circumstantial evidence, *e.g.*, its mineral nucleation capacity, phosphoprotein nature, large number of calcium binding sites, and its tight temporal and spatial tissue localization to mineralizing bone during development and in healing fractures (3, 6, 9, 10). However, no specific binding site has yet been identified at the gap region of native fibrillar type I collagen, a site at which initial mineral crystals were localized in a turkey tendon model (11).

As a means to investigate vesicle-mediated mineralization, we have studied biomineralization foci. Biomineralization foci (BMF) is discrete spherical extracellular structure produced by mineralizing osteoblastic cells in culture and *in vivo* within which initial mineral crystals are deposited inside membrane limited vesicles (12, 13). We have used the UMR106-01 rat osteoblastic cell line because the mineralization process within individual BMF in each culture is synchronized and occurs rapidly within 24 h after addition of phosphate to the cells (14). Our general hypothesis is that the protein composition of BMF is unique and reflects its functional capacity to produce hydroxyapatite crystals under cellular control. To test this hypothesis, we have focused our studies on defining the temporal sequence of events which precede mineral crystal deposition within BMF (14, 15) and on determining the protein composition of the BMF (15). For example, Raman confocal spectroscopic analysis of BMF at different times revealed that hydroxyapatite formation within individual BMF complexes is a multistep process. Specifically, changes in protein-derived signals at 1004 and 1660 cm^−1^ were found to reflect events which precede or accompany mineral crystal production because they can be blocked by protease inhibitor 4-(2-aminoethyl)benzenesulfonyl fluoride hydrochloride (AEBSF) (14). To test the concept that the protein composition of BMF is uniquely evolved for a role in mineral crystal formation, we isolated mineralized BMF with laser capture confocal microscopy (15). Consistent with this hypothesis, the results showed that the phosphoproteins bone sialoprotein and bone acidic glycoprotein-75 are preferentially localized to these structures. Furthermore, we showed that bone sialoprotein (BSP) is physically enriched on or in vesicles within biomineralization foci before mineral crystal formation (12). Interestingly, expression of these phosphoproteins temporally precedes mineral crystal formation and spatially demarks matrix sites *in vivo* and *in vitro* to which mineral crystals will be subsequently deposited. Recently, we have also demonstrated that BSP forms complexes with type XI collagen which can be immunoprecipitated from extracts of UMR106-01 osteoblastic cultures (16).

Type XI collagen is a minor fibrillar collagen widely distributed in tissues including cartilage, bone, and muscle, and its hereditary absence in Marshall’s syndrome (17), Stickler’s syndrome (18), fibrochondrogenesis (19), and nonsyndromic hearing loss deafness, autosomal dominant 37 (20) leads to facial and eye abnormalities, hearing loss, and joint problems. Although historically identified as distinct genes and distinct collagen types, the alpha chains of type V and XI collagens are now recognized to form heterotrimeric type V/XI triple helical collagen molecules (21, 22, 23). It is now appreciated that type V/XI collagen is required to nucleate and assemble type I and type II collagens into fibrils (23, 24). As a result, initial, small diameter type I fibrils observable in normal and pathologic cornea, bone, and cartilage are heterotypic structures representing hybrid structures composed of type I and type V/XI collagens (25). Current structural models of hybrid fibrils define a heterotypic alloyed core and a type I collagen outer sheath (26, 27). Importantly, these models place the retained N-terminal domain of the α1 chain of collagen type V/XI at the gap region on the fibril surface (28, 29).

The structure of the large N-terminal domain (NTD) domain of the Col11a1 chain is composed of a common shared N-propeptide (Npp) domain and a variable region (VR) domain subject to alternative exon splicing. Depending upon the tissue type and developmental stage, at least seven sequence variants are expressed with different combinations of the VR domain exons 6a, 6b, 7, and 8. It is noteworthy that four unique triplet lysine repeat sequences comprise structural motifs that are encoded within the “6b” exon. Because expression of the “6b” exon is restricted to bone, we hypothesized that these unique positively charged motifs present an extracellular binding site for acidic matrix phosphoproteins such as bone sialoprotein in bone. Thus, the goal of this project was to determine whether bone sialoprotein and Col11a1 interact specifically within the extracellular matrix and in/on osteoblastic cells in culture and in developing bone.

## Results

### Laser capture microdissection of mineralized biomineralization foci demonstrates selective enrichment in bone sialoprotein and type XI collagen but not dentin matrix protein 1

**Figure 1:**
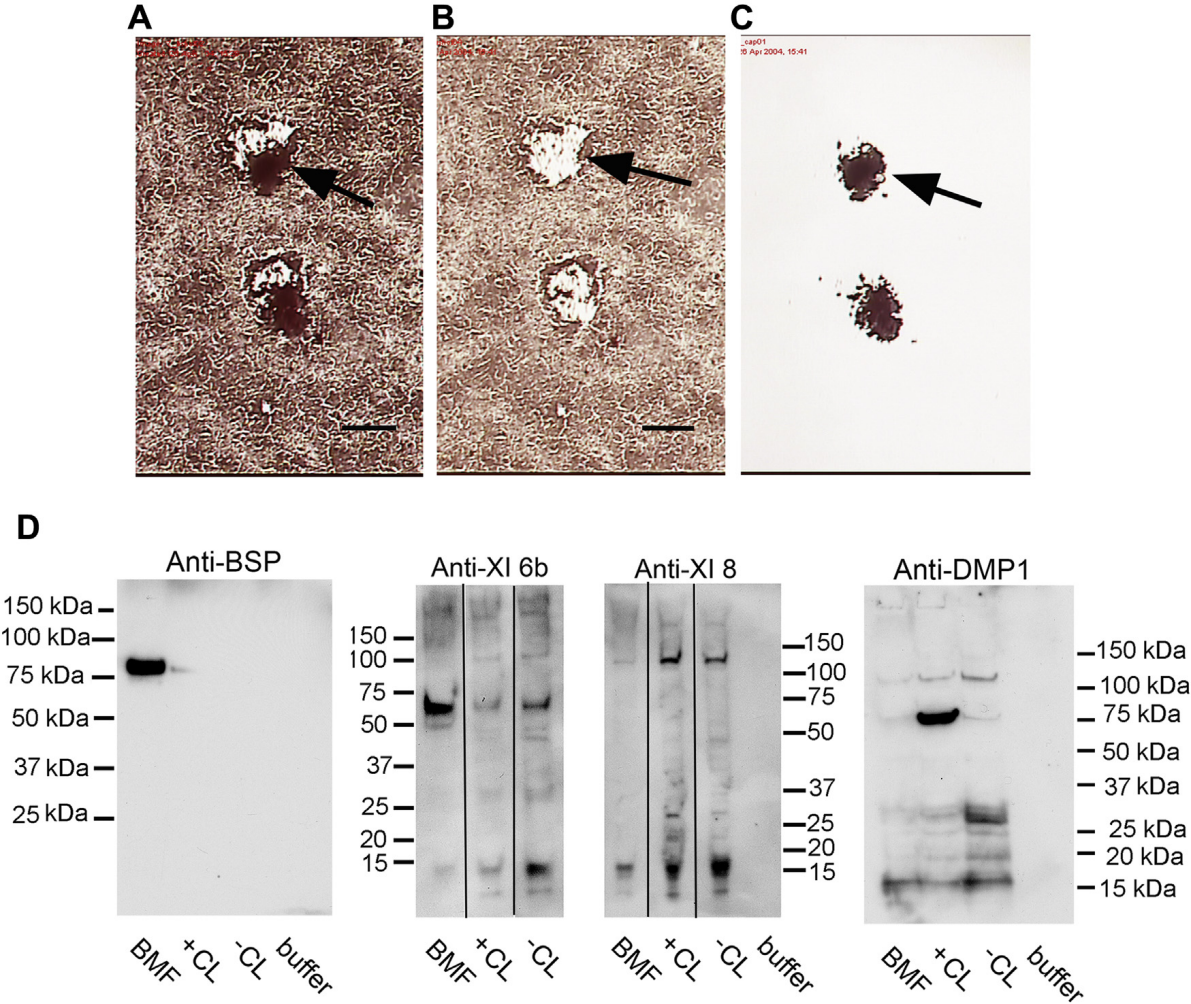
**Biomineralization foci are selectively enriched in bone sialoprotein and “6b” isoform of Col11a1 chain.***A*, alizarin red S stained biomineralization foci (*arrow*) before laser capture. Microscopic view of BMF before capture. Bar, 50 microns. *B*, view of same microscopic field shown in A after laser capture of two BMF. *Arrow* identifies position where uppermost BMF was removed. Bar, 50 microns. *C*, view of “cap” after laser capture of two BMF shown in *A*. *Arrow* identifies position of uppermost BMF captured. *D*, western blotting on pooled laser captured BMF sample compared with total mineralized cell layer (+CL), total unmineralized cell layer (−CL), and buffer alone (buffer). *Line*s between lanes represent splice junctures between different gel lanes electrophoresed on the same gel. Molecular weight estimates refer to blue prestained globular standards co-electrophoresed on the same gel. BMF, biomineralization foci.

We have shown previously that biomineralization foci are extracellular sites within osteoblastic cultures and in healing bone fracture tissue in which initial calcium hydroxyapatite crystals are deposited (12, 13). In a continuing effort to identify proteins which play a role in the mineralization process, we used laser capture microdissection to specifically isolate alizarin red–stained mineralized biomineralization foci (Fig. 1, *A*–*C*). Biomineralization foci (Fig. 1*C*) were preferentially separated from the cell layer (Fig. 1*B*). Isolated BMF (Fig. 1*C*) [(2000–3000)/preparation (∼60–80 μg protein)] were then dissociated in denaturing media and precipitated with acetone. Resultant samples were reduced and alkylated, split into equal parts, digested with trypsin, and double digested with trypsin/Glu-C protease and subjected to LC-MS/MS analysis as described in Methods.

**Figure 2:**
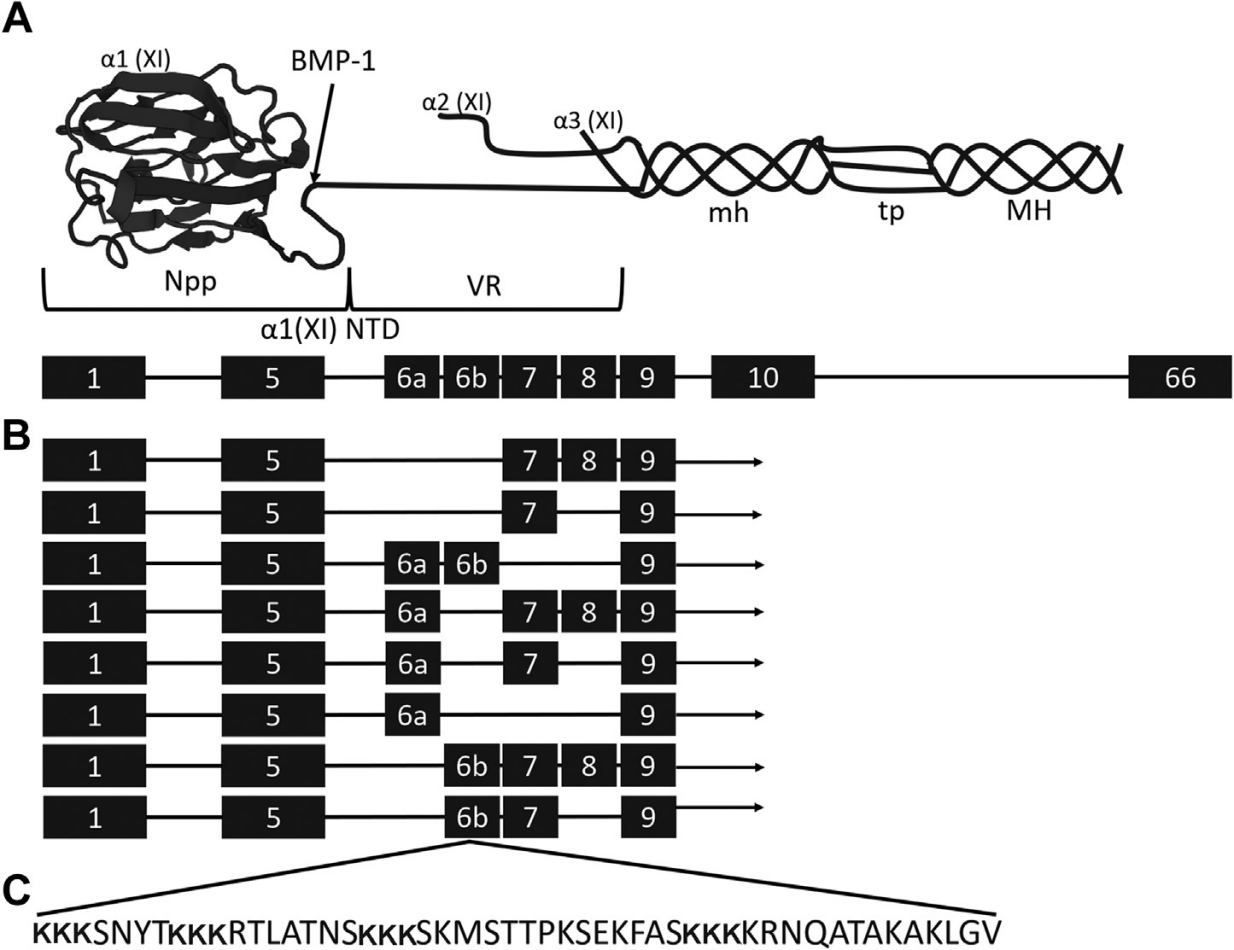
**Structural model of type XI collagen [adapted from Warner *et al.*** (30)**]**. *A*, type XI collagen is a heterotrimer of Col11a1, Col11a2, and Col11a3 of which Col11a1 contains a long N-terminal domain with constant (Npp) and variable sequence regions (VR). *B*, eight potential isoforms are expressed for the Col11a1 NTD region which depend upon alternative splicing of four different exons (6a, 6b, 7, 8). *C*, the 51-residue long 6b exon encodes four lysine triplet sequences. BMP-1, bone morphogenic protein 1 cleavage site; mh, minor helix; MH, major helix; Npp, N-propeptide; NTD, N-terminal domain; tp, telopeptide; VR, variable region.

Results of statistically significant peptide assignments based on these mass spectroscopic analyses are listed in Table 1. Interestingly, these assignments included bone sialoprotein, as well as the NTD of the Col11a1 chain. The NTD is an unusual extension preceding the collagenous, glycine-enriched, minor helix which, in contrast to other short-lived collagen propeptides, is at least partially retained within tissues. The NTD is composed of a shared Npp sequence (encoded by exons 1–5) and a variable VR sequence (encoded by exons 6a, 6b, 7, 8, and 9) (Fig. 2) (30). The two peptide sequences identified by LC-MS/MS are common to all seven alternatively spliced variants of Col11a1 NTD as part of the Npp region (Table 1 and Fig. 2).

**Table 1:** LC-MS/MS identification of peptides from two extracellular matrix proteins present in laser capture purified BMF from mineralizing UMR106-01 cultures

Protein (Mascot score)	UniProt Acc.#	Peptides (position)	Posttranslational modifications	Ions score/Expect	Enz.
Bone sialoprotein (458)	P13839	AEDSEENGVFK (28-38) KSSTVEYGEEYEQIGNEYNTAYETYDENNGEPR (257-289)	S4(phospho) N8(deamidated) N19(deamidated) N29(deamidated) T4(phospho)	57/1.2e-4	Tryp
		SSTVEYGEEYEQIGNEYNTAYETYDENNGEPR (258-289)	N18(deamidated) N28(deamidated)	82/4.5e-7	Tryp
		SSTVEYGEEYEQIGNEYNTAYETYDENNGEPR (258-289)	N18(deamidated) N28(deamidated)	90/6.1e-8	Tryp
		LAALQLPK (131-138)	T3(phospho) (unmodified)	51/6.3e-4	TRYP/GluC
Collagen alpha1 (XI) (343)	Q61245	RVSGSNEPNPVEEGFTEEYLTGEDYDVQR (322-350)	N6(deamidated)	107/1.5e-9	Tryp
		GVDGRDSDLLVDGDLGEYDFYEYK (362-385)	Q28(deamidated) (unmodified)	52/3.9e-4	Tryp

BMF, biomineralization foci.

To confirm that these proteins are localized to mineralization foci, extracts of laser captured BMF were compared by Western blotting with the same amount of protein/lane from extracts of total mineralized or unmineralized osteoblastic cell layer fractions. Under these conditions, a higher density band in BMF samples will reflect a quantitative enrichment. The results indicate that, on a relative basis, BSP protein is specifically enriched (∼10×) in the laser captured BMF as compared with the starting cell layer (Fig. 1*D*). When the type XI NTD “6b” or “8” specific epitopes were also probed, the former was also found to be enriched in the BMF sample as a 60 kDa fragment band while much lower amounts were evident in the +CL and −CL total extracts (Fig. 1*D*, middle panel). In contrast, epitope “8” (referring to exon 8) was not detectable in the BMF pool but was evident in both the +CL and −CL total extracts as a 110 kDa band. Finally, as a control, dentin matrix 1 was also probed to determine whether all acidic matrix phosphoproteins are localized to the laser captured mineralized biomineralization foci. Expression of dentin matrix protein1 is known to be increased upon exposure to beta-glycerolphosphate, which is added to +CL cultures to induce mineralization (Huffman and Gorski, unpublished result). However, it is evident that dentin matrix protein1, while strongly expressed as a monomeric band at 75 kDa within the cell layer of mineralized osteoblastic cultures, is not localized within BMF (Fig. 1*D*, right panel). In view of the co-localization of BSP and the 60 kDa “6b” containing NTD domain of type XI collagen within biomineralization foci, we then asked whether these two proteins may interact with each other.

### Co-purification of BSP and NTD domain of Col11a1 collagen upon ion-exchange chromatography

**Figure 3:**
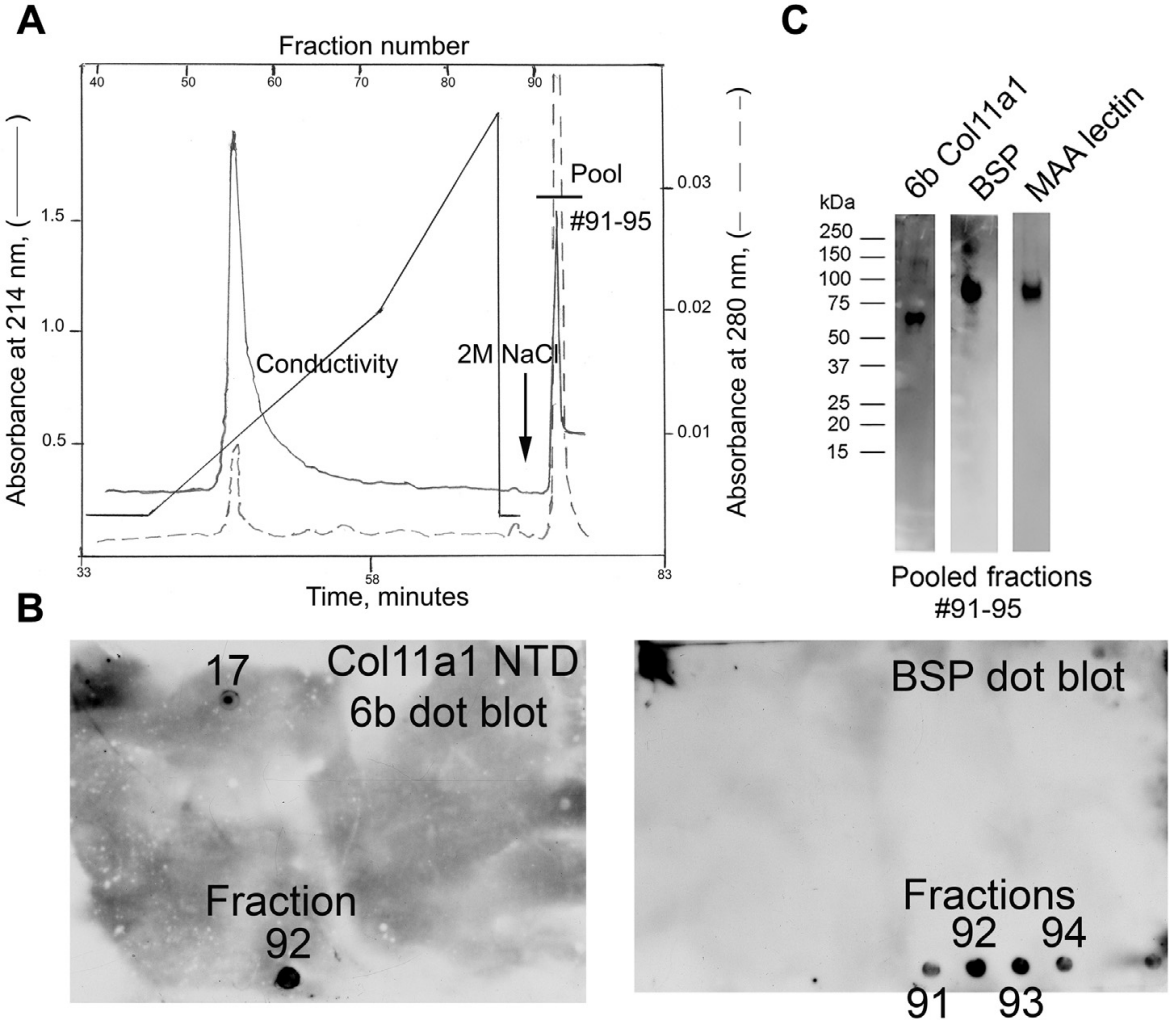
**Co-purification of 60 kDa Col11a1 NTD “6b”-expressing fragment with full-length bone sialoprotein by anion-exchange chromatography.***A*, tracing of anion-exchange chromatography of EDTA extract of mineralizing UMR106-01 osteoblastic cultures. Flow-thru fractions are not shown before the start of the linear 0 to 0.6 M sodium chloride salt gradient. UV readings at 214 and 280 nm, and conductivity are plotted as a function of time and fraction number. At the end of the salt gradient, a 2 M NaCl step gradient was applied (designated by *arrow*). *B*, individual fractions were dot-blotted sequentially onto PVDF membrane and subjected to immunodetection with antibodies against Col11a1 chain NTD “6b” epitope or bone sialoprotein. *C*, peak fractions eluting with 2 M NaCl (#91–95) were pooled and subjected to SDS-PAGE, and the gel was either stained for MAA lectin, which identified a prominent BSP band at 90 kDa, or, the gel was western blotted with anti-BSP and anti-Col11a1 NTD “6b” epitope specific antibodies. Molecular weight estimates refer to blue prestained globular standard proteins co-electrophoresed on the same gel. BSP, bone sialoprotein; MAA, Maackia amurensis agglutinin; NTD, N-terminal domain; PVDF, polyvinylidene difluoride.

We have showed previously that the contents of mineralized biomineralization foci can be selectively extracted from UMR106-01 cultures with 50 mM EDTA, pH 7.5 (15). To further analyze the binding capacity of BSP for the Col11a1 NTD domain, we subjected these EDTA extracts to anion-exchange chromatography at pH 5.2. Under these conditions, it is predicted that cationic proteins like the 60 kDa NTD fragment would not bind to the resin and should be eluted directly. Data representative of triplicate runs are depicted in Figure 3*A*, where following sample application, proteins were eluted with a gradient of from 0.05 to 0.6 M NaCl followed by final step gradient with 2 M NaCl as a limit solvent. Column flow was monitored continuously for protein absorbance (214 and 280 nm) and conductivity, and equal aliquots of individual fractions were subjected to dot blotting with anti-BSP or Col11a1 NTD “6b” epitope specific antibodies (Fig. 3*B*). As evidenced by the UV-absorbance tracings, the majority of proteins in the EDTA extract eluted at about 0.2 M NaCl during the salt gradient. A sharp peak was also observed to elute with the 2 M NaCl step gradient (Fig. 3*A*). Dot immunoblotting of each fraction revealed several interesting points (Fig. 3*B*). BSP and “6b” epitope immunoreactivity both eluted predominantly with the 2 M NaCl step gradient. Based on staining, >80% of the “6b” immunoreactive 60 kDa fragment was found to co-elute with BSP in fractions #91 to 95 (compare Fig. 3, *A* and *B*). This conclusion is confirmed by Western blotting after fractions #91 to 95 were pooled for further analysis. Specifically, the pooled 2M NaCl fractions contain both full length BSP (90 kDa) and a 60 kDa Col11a1 “6b” immunoreactive NTD fragment (Fig. 3*C*). Maackia amurensis agglutinin lectin staining, which is restricted to BSP, also confirms its enrichment in the 2.0 M NaCl peak (Fig. 3*C*). Taken together, results in Figure 3 indicate that full length BSP is able to bind tightly to the Col11a1 NTD domain, *e.g.*, resisting dissociation in high salt concentrations (>0.6 M NaCl).

### Comparative binding of Col11a1 NTD VR-derived “6a” or “6b” peptides to osteoblastic cells

To quantitatively evaluate the relative sequence specificity of the BSP/Col11a1 NTD binding interaction, we carried out direct binding studies with a series of peptides representing parts of the variable region of the alternatively spliced NTD sequence. An inherent limitation associated with peptide studies is that they may not faithfully reproduce their native conformations. We focused our attention on exon 6 because the “6b” epitope was localized to BMF extracts along with BSP (Fig. 1*D*) and contained four lysine triplet sequences which appear to be a unique motif restricted to Col11a1 based on NCBI protein BLAST searches (Gorski, data not shown). Specifically, we tested three overlapping peptides derived from the 51-residue “6b” exon sequence (peptides 3, 4, and 5) and compared them with two “6a” exon-derived peptides which are themselves enriched in acidic amino acid residues (peptides 1 and 2) (Fig. 2 and Table 2). “6a” and “6b” represent competing choices during alternative splicing of the NTD domain and are not expressed in the same NTD domain. Peptides tested were each N-terminally conjugated with biotin to facilitate fluorescent detection.

**Table 2:** List of Col11a1 chain NTD-derived peptides used in binding studies (N terminally conjugated with FAM or biotin)

PEPTIDE 1: biotin- or FAM-N-YAPEDIIEYDYEYGETDY**K** (6a derived peptide)
PEPTIDE 2: biotin- or FAM-N-EAESVTEMPTVTEETVAQTE (6a derived peptide)
PEPTIDE 3: biotin- or FAM-N-**KKK**SNYT**KKK**RTLATNS**KKK**S**K**M (6b derived peptide)
biotin- or FAM-N-**KKK**SNYT**KKK**RTLATNS**KKK** (both versions gave similar results)
PEPTIDE 4: biotin- or FAM-N-STTP**K**SE**K**FAS**KKKK**RNQASA**K**A**K** (6b derived peptide)
PEPTIDE 5: biotin- or FAM-N-**KKK**S**K**MSTTP**K**SE**K**FAS**KKKK**R (6b derived peptide)

UMR106-01 osteoblastic cells were grown to confluence under serum depleted conditions in multiwell plates under mineralizing (with β-glycerolphosphate [BGP]) or nonmineralizing conditions (without BGP) and binding studies carried out with monolayer cultures fixed at different times after addition of mineralization inducer BGP. Prior publications with the UMR106-01 model show cells within each culture become temporally synchronized and progressively differentiate starting at 64 h after plating (when BGP is added) up to 82 h when calcium hydroxyapatite crystals start forming within biomineralization foci (14, 15, 31). Peptide binding was carried out in serum-free medium containing 10 μg/ml peptides #1 to #5, and bound peptides were detected with rhodamine-conjugated streptavidin using a fluorescence plate reader.

**Figure 4:**
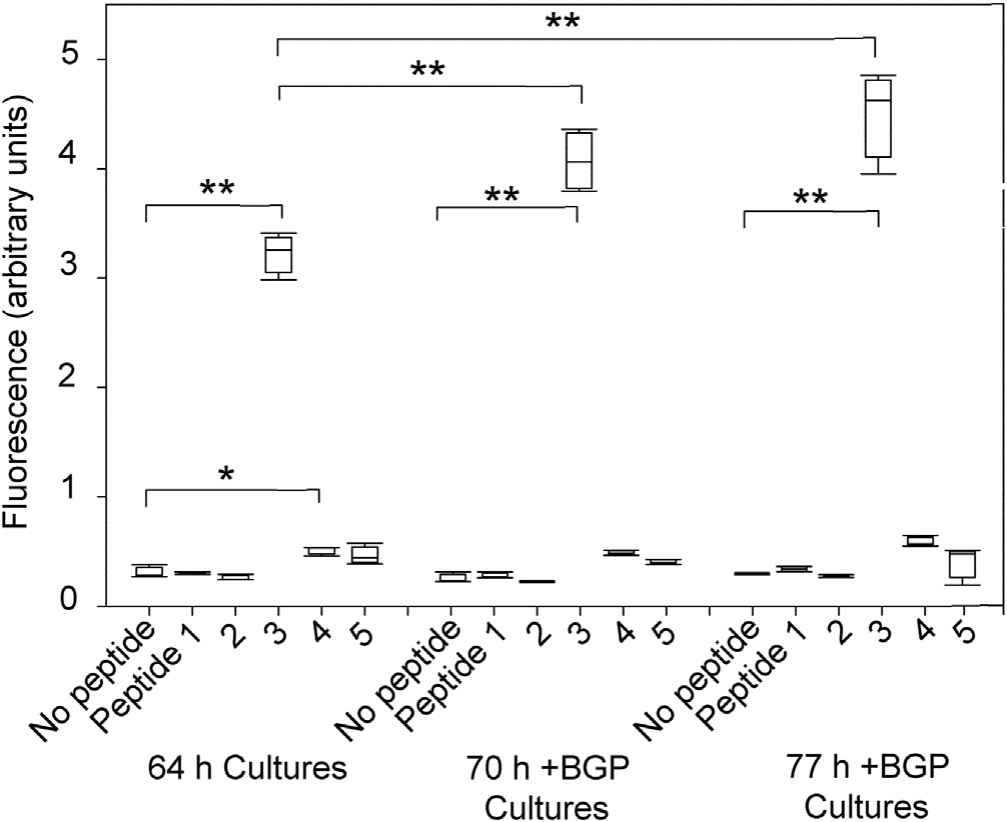
**Binding of “6a-“ and “6b”exon-derived peptides to mineralizing osteoblastic cells depends upon the number of lysine triplet motifs.** Box scatter plot depicting binding of peptides where the dimensions of the boxes represent the first and third quartiles and the lines through the boxes the median. UMR106-01 osteoblastic cells were plated, grown, and differentiated as described in Methods, and stopped with 70% ethanol at 64, 70, and 77 h after plating. Cultures were then rehydrated with 0.05 M Hepes buffer (pH 7.5), containing 0.15 M sodium chloride, and the endogenous biotin content of cultures was blocked with streptavidin/biotin solutions (Vector, Inc). N-terminal biotin-labeled Col11a1 chain NTD peptides (#1–5) (10 μg/ml) were dissolved in 0.05 M Hepes buffer (pH 7.5), containing 0.15 M sodium chloride and 1 mg/ml casein and incubated with the fixed cells overnight. Free peptide was removed by multiple washing steps, and bound peptides were detected by incubation for 1 h with rhodamine-labeled streptavidin. Following multiple washes, the cells were scraped off the culture wells into a small volume of buffer, and the suspension was transferred to a 96-well white microtiter plate. Bound rhodamine was quantitated in a fluorescence plate reader. Peptide binding studies were carried out in quadruplicate and values analyzed statistically as described. Error bars = STD and *asterisks* refer to significant comparisons with the no peptide control or with the 64 h time point as noted. ∗*p* < 0.01; ∗∗*p* < 0.001. BGP, β-glycerolphosphate; NTD, N-terminal domain.

As shown in Figure 4, only peptide 3, which represents a part of the “6b” exon containing three lysine triplet sequences, consistently displayed robust binding to the cell layer at all times and conditions. Peptide 3 binding to cells was greater than any of the other peptides at any culture time (*p* < 0.001). Interestingly, binding for peptide 3 increased only about 1.4× from 64 h to 77 h +BGP (*p* < 0.001), which is consistent with cDNA array analyses which show that BSP mRNA levels remain relatively constant over this time period (Chittur and Gorski, unpublished result) (Fig. 4). In contrast, binding of peptides 1 and 2, which represent parts of the “6a” exon, to cells was generally indistinguishable from that for the no peptide control. However, peptides 4 and 5, which represent parts of the “6b” exon containing 1 and 2 triplet lysine sequences, respectively, did exhibit quantitatively higher, but not significantly different, binding than the no peptide control at the 77 h +BGP time point (Fig. 4). Taken together, these results suggest that biomineralization foci within osteoblastic cell layers display a distinct preference for binding peptide 3 which contains three lysine triplet sequences as opposed to peptides 4 and 5 which contain fewer such motifs (Figs. 2 and 4).

### COL11a1 NTD “6b” exon-derived N-labeled 6-carboxyfluorescein-peptide 3 binds specifically to BSP and nucleolin in extracts from mineralizing osteoblastic cultures

**Figure 5:**
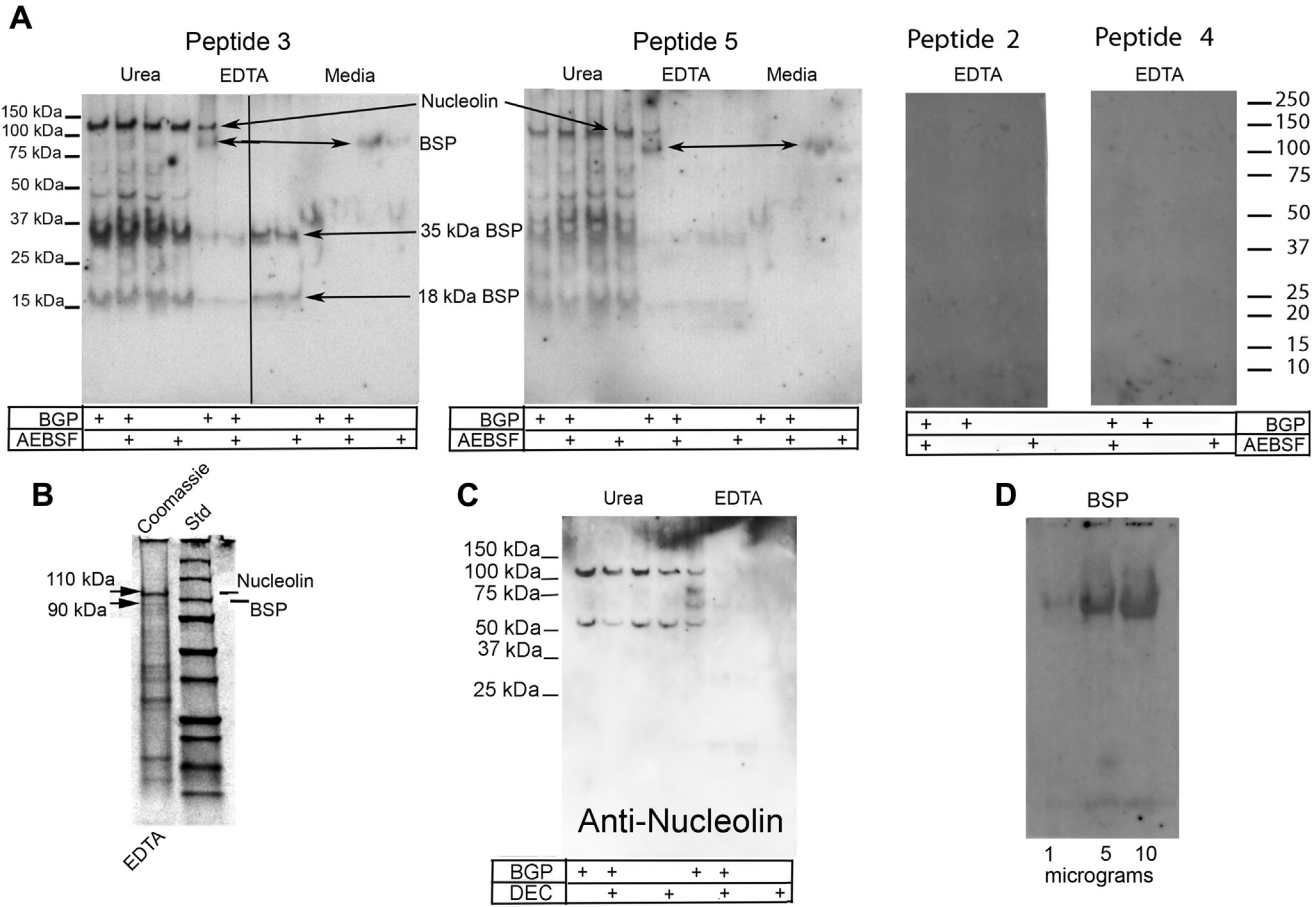
**Col11a1 peptide 3 binding to osteoblastic cell fractions identifies two protein ligands: bone sialoprotein and nucleolin.***A*, western blots identify bone sialoprotein and nucleolin. “12 pattern” SDS PAGE gel was run as noted previously (15, 31), subjected to Western blotting, and blots incubated with FAM-labeled peptide 3. Lanes represent four different cell culture conditions [mineralizing (+BGP) and nonmineralizing (−BGP, +BGP + AEBSF protease inhibitor, −BGP + AEBSF)], three different cell fractions from each condition [(cell media, EDTA extract enriched in biomineralization foci), and urea extract (cell membrane and contents)]. Line on figure indicates position of splice junction between gel lanes electrophoresed on the same gel. Molecular weight estimates are based on blue prestained globular protein standards co-electrophoresed on the same gel. *B*, gel lanes after electrophoresis of EDTA extract provides material for LC-MS/MS peptide mapping and Edman sequencing. EDTA extract from mineralizing UMR106-01 cultures electrophoresed on SDS-PAGE gel and stained with Coomassie brilliant blue dye. Bands at 110 kDa and 90 kDa were excised (*arrows*) and subjected to mass spectroscopic peptide mapping. A band at 18 kDa (*arrow*) was also cut out and the contents subjected to micro-Edman sequencing. Standard lane (Std): 250 kDa, 150 kDa, 100 kDa, 75 kDa, 50 kDa, 37 kDa, 25 kDa, 20 kDa, and 15 kDa. See Methods for more details. *C*, western blot incubated with anti-nucleolin antibodies. Urea and EDTA extracts from mineralizing UMR106-01 cultures were electrophoresed on SDS-PAGE gel and blotted onto PVDF membrane. Lanes represent four different cell culture conditions [mineralizing (+BGP) and nonmineralizing (−BGP, +BGP + DEC protease inhibitor, −BGP + DEC inhibitor)]. Molecular weight estimates are based on blue stained globular protein standards co-electrophoresed on the same gel. *D*, purified full length calvarial bone sialoprotein binds Col11a1 NTD derived peptide 3 robustly. Bone sialoprotein was purified from calvarial bone, subjected to SDS-PAGE over a range of 1 to 10 μg protein/lane, and blotted onto PVDF membrane as described in Methods. Blots were blocked with casein and then incubated with FAM-labeled Col11a1 NTD derived peptide 3. After excess peptide was removed by washing, blots were imaged with a Fuji LS 4000 fluorescent imager. +B, plus BGP; +B + DEC, plus BGF and decanoyl-RRLL-chloromethylketone; +DEC, without BGP and with decanoyl-RRLL-chloromethylketone; and, blank, without BGP and without decanoyl-RRLL-chloromethylketone; AEBSF, 4-(2-aminoethyl)benzenesulfonyl fluoride hydrochloride; BGP, β-glycerolphosphate; BSP, bone sialoprotein; FAM, 6-carboxyfluorescein; NTD, N-terminal domain; PVDF, polyvinylidene difluoride.

In view of the strong preference of peptide 3 binding to UMR106-01 cells, we next addressed the identity of the protein ligand or ligands to which it was binding. Briefly, we carried out binding studies in which N-6-carboxyfluorescein (FAM)–labeled peptides representing sequences #2, 3, 4 or 5 sequence (Table 2) were incubated individually with polyvinylidene difluoride (PVDF) membrane blots containing proteins extracted from UMR106-01 osteoblast-like cells. Blots were made with up to three different cell fractions representing in total the entire culture, *e.g.*, media, EDTA extracts of BMF, and 8M urea extracts of the cell layer. Each cell fraction was itself derived from four different culture conditions (+BGP; +BGP +AEBSF; −BGP; −BGP +AEBSF) where each was electrophoresed side by side and incubated with peptide (Fig. 5). Blots were then washed extensively with buffer to remove free peptide before scanning with a fluorescence imager. Similar to binding results with fixed UMR106-01 cultures above, positive binding was observed primarily with peptide 3 (Fig. 5*A*), although similar weaker results were also noted with peptide 5 albeit with less uniformity. No bands were detected with peptides 1 (Huffman and Gorski, data not shown), 2, or 4 in any experiments. For simplicity, we only show the results with EDTA extracts for these latter peptides (Fig. 5*A*).

The pattern of peptide 3 binding was largely the same for all urea extracts which comprise predominantly intracellular and membrane proteins. Specifically, three major protein bands were detected at 110 kDa, 35 kDa, and 18 kDa. For EDTA extracts, which contain proteins largely derived from biomineralization foci (15), binding results were dramatically different depending upon culture conditions (Fig. 5*A*). Under mineralizing conditions (+BGP), two bands predominated at 110 kDa and at 90 kDa, while these bands were missing when the cultures were also treated with mineralization inhibitor AEBSF (+BGP +AEBSF). In the absence of BGP, the larger bands were absent, but instead two additional bands were observed at 35 kDa and at 18 kDa (−BGP). Finally, the media fraction was largely devoid of proteins binding peptide 3 except for cultures not treated with either BGP or AEBSF where a band at 90 kDa was evident (−BGP, Fig. 5*A*).

In an effort to identify the proteins binding to peptide 3, the EDTA extract from mineralizing cultures (+BGP) was electrophoresed, and after staining with Coomassie blue dye, bands at 110 kDa and 90 kDa were excised (Fig. 5*B*). These bands along with control slices were subjected to mass spectroscopic peptide mapping. Results permitted the following two assignments: nucleolin as the 110 kDa band (based on two peptides: GYAFIEFASFEDAK and FGVFESAEDLEK) and bone sialoprotein or heat shock protein HSP-90 as the 90 kDa band (based on peptide HAYFYPPLK or HFSVEGQLEFR, respectively). In addition to mass spectroscopic peptide mapping, a Coomassie Blue stained band at 18 kDa was excised and subjected to micro-Edman protein sequencing (Fig. 5*B*). The resultant partial sequence, N-FSMKNFHRRIKA, was identical to the N-terminal sequence for rat bone sialoprotein (32).

Western blotting of UMR106-01 cell fractions confirmed the presence of nucleolin (Fig. 5*C*). Briefly, nucleolin was predominantly localized to urea extracts which are enriched with intracellular proteins and cell membranes (15). Antinucleolin antibodies recognized the full-length 110 kDa isoform in all urea extracts from all four culture conditions, as well as in the EDTA extract of +BGP cultures enriched in the contents of biomineralization foci. However, repeated attempts to detect smaller nucleolin fragments within UMR106-01 cell fractions were largely unsuccessful (Fig. 5*C*).

Because results presented in Figure 5, *A* and *B* identified BSP as the 90 kDa protein which binds peptide 3, we asked whether purified bone sialoprotein purified from rat calvarial bone would also bind Col11a1 chain NTD-derived N-FAM-labeled peptide 3. After electrophoresis and blotting, strong positive binding was detected with from 1 to 10 μg of full length bone sialoprotein (Fig. 5*D*). By comparison, 10 μg of a related SIBLING rat phosphoprotein, osteopontin, was undetectable (Gorski, data not shown).

### Confocal imaging of N-FAM–labeled peptide 3 binding to osteoblast-like cells

**Figure 6:**
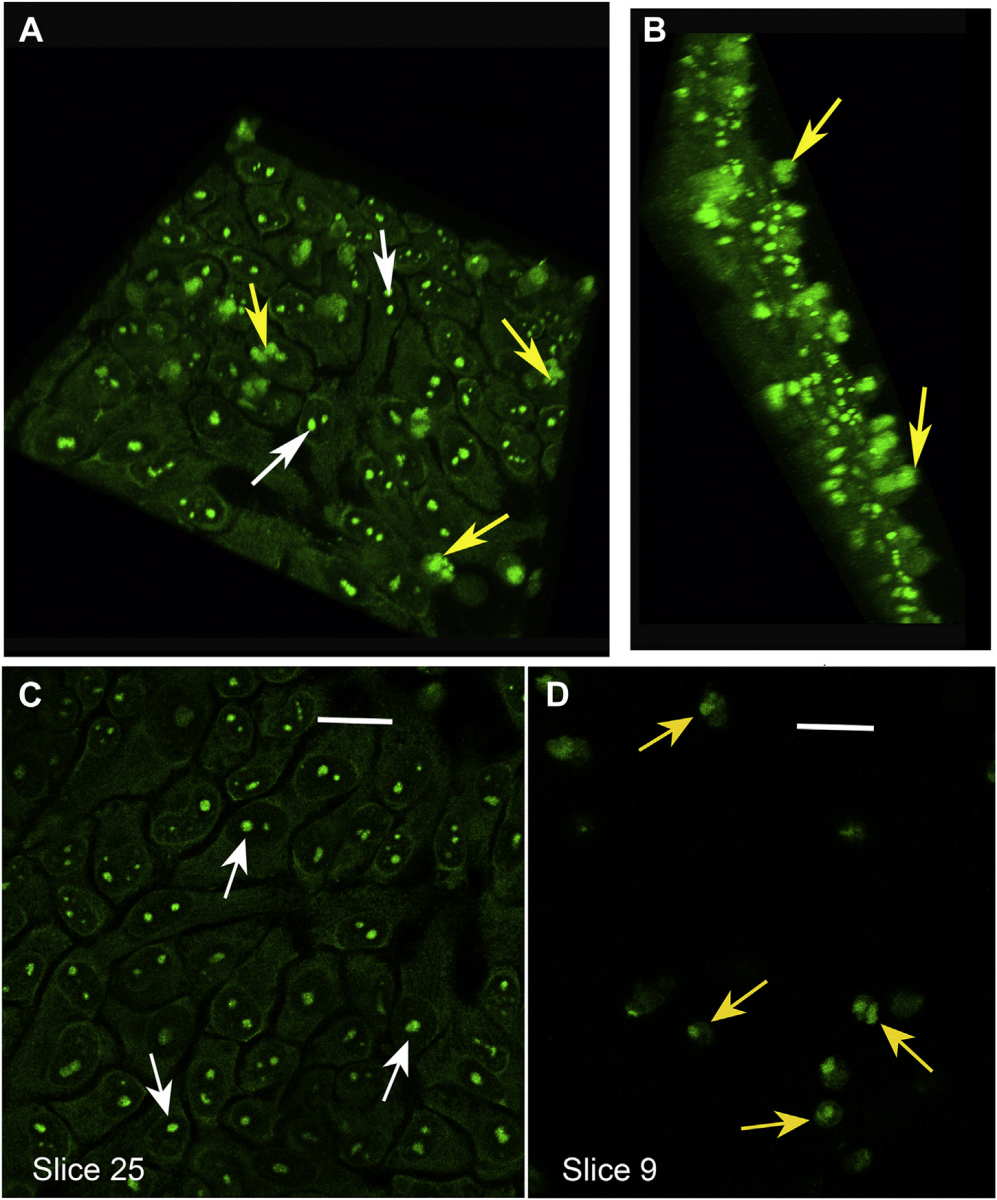
**FAM-labeled peptide 3 binding identifies nucleoli and apparent vesicular binding sites within biomineralization foci in osteoblastic cultures.** UMR106-01 osteoblastic cells were plated and grown and differentiated as described in Methods. At 64 h after plating, mineralization was induced by addition of BGP and cultures were stopped by mild fixation with 70% ethanol at 77 h. FAM-labeled peptide 3 (10 μg/ml) was incubated with cell monolayers overnight and then, after extensive washing, visualized by confocal microscopy with a 40× oil immersion lens. *A*, compressed z-stack partially rotated view of cell monolayer showing labeled nucleoli (*white arrows*). *B*, compressed z-stack side view of cell monolayer illustrating the projection of labeled spherical biomineralization foci (*yellow arrows*) above the plane of the cells. *C*, selected *en face* view showing plane containing cell monolayer with FAM-labeled peptide 3 binding to 1 to 2 nucleoli within each nuclei (*white arrows*). Scale bar = 200 microns. *D*, selected *en face* view showing plane containing multiple biomineralization foci above the cells (see *B* above) which demonstrates FAM-labeled peptide 3 binding to apparent spherical vesicles within them (*yellow arrows*). Note that views *C* and *D* are taken from the same z-stack and each align/overlap starting from the left edge. Scale bar = 200 microns. BGP, β-glycerolphosphate; FAM, 6-carboxyfluorescein.

In view of the preference of Col11a1 NTD peptide 3 for binding to bone sialoprotein and to nucleolin, we next carried out confocal microscopic imaging to determine the distribution of binding sites within UMR106-01 osteoblastic cells. Monolayer cultures were prepared as described above and briefly fixed with ethanol before the time at which biomineralization foci become mineralized. Imaging of Col11a1 chain NTD binding sites was achieved by addition of N-terminally labeled FAM-labeled peptide 3. Confocal microscopic images of several views of the resultant labeled monolayers are shown in Figure 6. In a condensed z-stack rotated view, one or more brightly stained spherical nucleoli (white arrows) are clearly evident internally within each cell (Fig. 6*A*). Less distinct, but also brightly stained, are multilobulated biomineralization foci which are marked with yellow arrows. The fact that biomineralization foci project out and away from the surface of the cell monolayer (12, 13) is demonstrated in a condensed z-stack side view (Fig. 6*B*). Closer analysis of individual slices within the z-stack reflecting the plane of individual BMF reveals that the fluorescent signal within each is organized into smaller spherical structures (yellow arrows) about 5 microns in diameter (for example, slice 9, Fig. 5*D*). These spherical structures appear similar in size and shape to confocal Raman spectroscopic images of the first mineral crystal deposits within biomineralization foci in synchronized UMR106-01 cultures (14). Individual nucleoli (white arrows) are also clearly visible when the plane of section selected is lowered (slice 25, Fig. 5*C*). Based on the documented enrichment of nucleolin within nucleoli and the prior TEM immunogold localization of BSP to large vesicles within BMF (13), we conclude that visualization of nucleoli and biomineralization foci with FAM-labeled Col11a1 NTD “6b-derived” peptide 3 is due directly to binding to these two proteins, respectively.

### Peptide 3 binds specifically to three skeletal sites known to be enriched in bone sialoprotein

**Figure 7:**
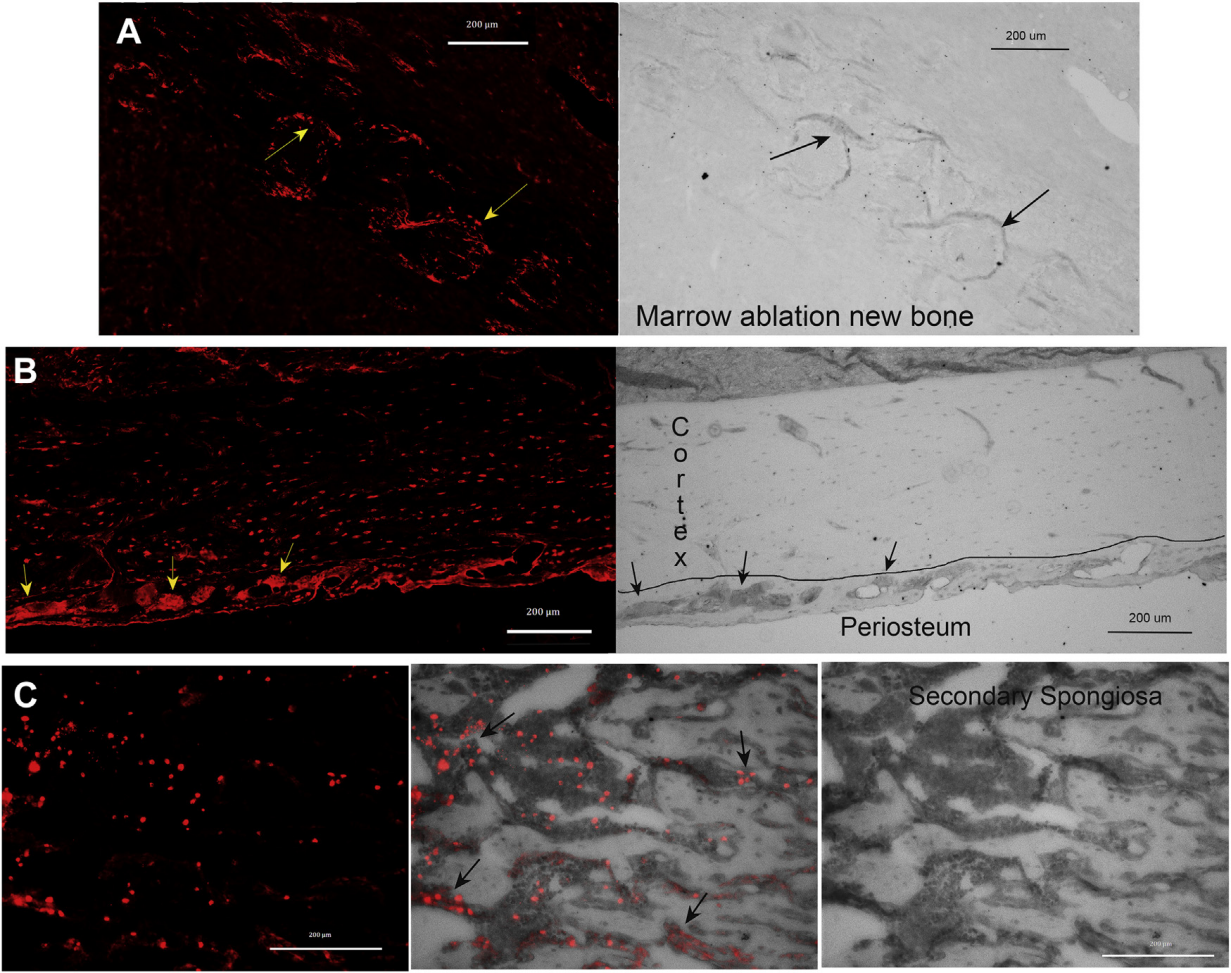
**Peptide 3 binds specifically to three skeletal sites known to be enriched in bone sialoprotein.** Biotin-labeled peptide 3 was imaged after binding to sections of decalcified bone tissue (see Methods) by detection with rhodamine-conjugated streptavidin; controls with peptide 1 were negative and below the limits of detection in all cases (not shown). *A*, fluorescent and brightfield images of decalcified section of very early new intramembranous bone. At day 4 in fracture healing tibial marrow ablation model in young rats, small semicircular dense areas of new osteoid form within the preexisting fibrin clot (*arrows*). Each of these condensed regions stains strongly with peptide 3 (*arrows*). For reference, we have shown previously the first mineral deposited within the ablated region peaks at day 6 to 7 within similar regions of condensed osteoid (40). *B*, fluorescent and brightfield images of young rat periosteum and bony cortex. Densely staining osteoblastic cells visible in the brightfield image of the basal periosteal layer (*arrows*), which strongly express bone sialoprotein (13), bind peptide 3 robustly (*arrows*). Many osteocytes within the cortex also bind peptide 3 weakly. *Black line* demarks the approximate border of the basal periosteum and mineralized cortical bone. *C*, fluorescent, overlay, and brightfield images of young rat secondary spongiosa. Peptide 3 binds primarily to cells within or closely associated with condensed osteoid regions of the secondary spongiosa (*arrows*). Individual scale bars are shown on their respective images.

We then asked whether peptide 3 would bind specifically to bone sialoprotein within its native bony environment. Briefly, N-terminal biotin labeled peptide 3 was incubated overnight with sections of paraffin-embedded decalcified bone tissue, and then, bound peptide was visualized with rhodamine-conjugated streptavidin (Fig. 7). To investigate peptide 3 binding in a variety of skeletal sites, we looked at a fracture healing model and the tibia in rats. As illustrated in Figure 7*A*, peptide 3 bound specifically to sites of very new bone formation at day 4 in the marrow ablation fracture healing model. Specifically, peptide 3 bound to thin condensed matrix regions that form *de novo* within the fibrin clot that fills the tibial marrow space at day 4 after ablation (Fig. 7*A*, arrows). These staining patterns resemble in size and shape “mineralization centers” which represent the first step of intramembranous bone formation. Controls with biotin-labeled peptide 1 yielded negative results (Gorski, data not shown). Similar to other sites of intramembranous bone formation, *e.g.*, embryonic calvarium, bone sialoprotein is known to be one of the first noncollagenous matrix proteins localized to these thin slivers of forming bone (33, 34). When the tibial boney collar was also examined, biotin-labeled peptide 3 bound specifically to densely appearing osteoblastic and preosteocytic cells within the basal region of the periosteum (Fig. 7*B*, arrows). We have shown previously that bone sialoprotein is expressed strongly in similar cells at this same site (13). Secondarily, weaker peptide 3 binding to embedded osteocytes was also observed within the bony cortex (Fig. 7*B*). However, controls with peptide 1 gave results which were all below the level of fluorescent detection. As shown in Figure 7*C*, peptide 3 also bound specifically to cells either within or closely associated with the tibial secondary spongiosa. For ease of comparison, the image of fluorescent peptide 3 binding is overlaid upon the brightfield image of the secondary spongiosa (Fig. 7*C*). Consistent with this binding, bone sialoprotein has been reported to be enriched at this skeletal site (35). In contrast, at all of the above skeletal sites, peptide 3 binding to cells and matrix within adjacent muscle and connective tissue was negligible.

### Anti-BSP antibodies and N-biotinylated-peptide 3 both co-localize to extracellular matrix and cells within newly forming bone

**Figure 8:**
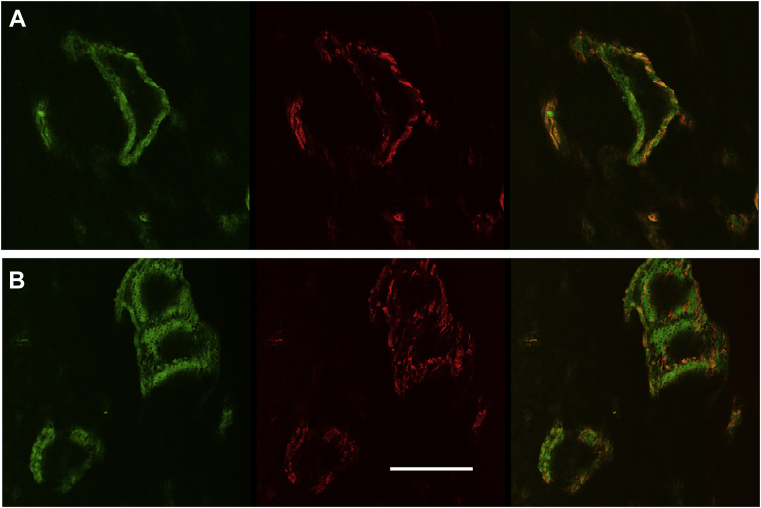
**Both anti-BSP antibodies and N-biotinylated-peptide 3 co-localize to the same extracellular matrix sites and cells within newly forming bone.***A* and *B*, co-labeling of bone sialoprotein with biotinylated peptide 3 and monospecific antibone sialoprotein antibodies (representative *red channel*, *green channel*, and overlay views are shown). New healing bone was prepared using a rat tibial marrow ablation model (40), and tibias were removed 4 days after surgery, fixed, decalcified, embedded in paraffin, and sectioned lengthwise before analysis. Anti-BSP antibody staining (*green*) of bone sections followed an indirect detection method using Alexa 488 conjugated goat secondary antibodies, and biotinylated peptide 3 (*red*) was visualized with Alexa 594-conjugated streptavidin as described in Methods. Images were obtained using confocal microscopy with a 20× objective. Scale bar, 50 microns, applies to both *A* and *B*. BSP, BSP, bone sialoprotein

In view of the apparent robust staining observed for biotinylated-peptide 3 in bone tissue (Fig. 7), we sought to confirm the specificity of binding by carrying out double labeling with antibone sialoprotein antibody and biotinylated-peptide 3 simultaneously. To relate the double labeling to prior results in Figure 7, we used adjacent consecutive sections of day 4 marrow ablated whole rat tibias used previously. Concurrent indirect immunolabeling using anti-BSP antibodies and Alexa 488–conjugated secondary antibodies (green) was carried out along with incubation with biotinylated peptide 3 and Alexa 594 conjugated Streptavidin. Strong staining was achieved with both labeling methods (Fig. 8, *A* and *B*). Importantly, antibone sialoprotein primary antibodies and biotinylated peptide 3 were both found to co-localize closely to extracellular matrix and cells within newly forming bone. Specifically, in Figure 8*A*, green antibone sialoprotein immunostaining was observed to form an evenly labeled matrix layer defining a roughly spherical shape with a hollow center. Remarkably, the width of the wall of the spherical structure was relatively constant throughout its length. At the same time, biotinylated-peptide 3 patterned the outlines of this antibody staining closely while appearing to label regions of the spherical structure more strongly than others (Fig. 8*A*). A second representative field is depicted in Figure 8*B*, where two connected spherical structures label strongly with both anti-BSP antibodies and peptide 3. Again, green antibone sialoprotein labeling was found to rather uniformly stain an outer layer of matrix while a central hole or cavity was left unlabeled. Biotinylated peptide 3 labeling closely follows that for the outlines of anti-BSP immunostaining. However, as observed in Figure 8*A*, peptide 3 labeling is not as uniform as that with antibody. Rather, peptide 3 seems to display both strong punctate (cellular) staining as well as weaker more homogeneous matrix staining. At this resolution, it is not possible to determine whether peptide 3 is binding to the surface of osteoblastic cells or at internal sites. Based on these results, we conclude that biotinylated peptide 3 labeling of tibial bone tissue and healing new bone co-localizes closely with bone sialoprotein on/in osteoblastic cells and matrix as defined with monospecific antibodies.

## Discussion

The data presented here identify for the first time a specific binding interaction between bone sialoprotein and a unique lysine-triplet–enriched “6b” alternatively spliced exonal sequence of the NTD of Col11a1. Several key experimental results support this conclusion. First, comparative Western blotting of osteoblastic culture fractions demonstrates that biomineralization foci, the contents of which are preferentially extracted by 50 mM EDTA, are enriched in 110 kDa and 60 kDa N-terminal fragments of Col11a1 containing this lysine-triplet enriched sequence. Polypeptides containing a different alternatively spliced variant “6a” exonal sequence were not similarly co-localized. We have defined biomineralization foci as supramolecular extracellular matrix structures which are the sites of initial hydroxyapatite crystal deposition in UMR106 to 01 osteoblastic cultures (13) and in forming intramembranous bone (12) where these sites are also termed “mineralization centers”. Second, when extracted from osteoblastic cultures with 8 M urea and 0.05 M EDTA, bone sialoprotein was found to co-purify during anion exchange chromatography with a 60 kDa N-terminal fragment of Col11a1 containing the “6b” sequence. Dissociation of the two proteins required exposure to 2 M NaCl. Under these conditions, the basic charge of the 60 kDa Col11a1 NTD fragment should have precluded its binding to the anionic resin at pH 5.2, let alone necessitating 2.0 M salt for elution. Importantly, these results confirm our prior immunoprecipitation results which showed that full length 90 kDa BSP co-purified with a 60 kDa cationic protein that cross-reacted with “6b” antibodies (36). Third, specific binding of the Col11a1 NTD 51-residue long “6b” exonal sequence to bone sialoprotein was demonstrated indirectly with overlapping shorter peptides. Specifically, peptide 3, which contains three lysine triplet sequences, showed the greatest quantitative binding while comparable peptides enriched in lysine residues and containing less than three lysine triplets or derived from the Col11a1 NTD “6a” exonal sequence gave largely background binding. Fourth, a similar binding site was separately identified for nucleolin which is known to bind a lysine-enriched nucleolar localization signal sequence (37). Fifth, the specificity of peptide 3 binding was further illustrated upon addition of FAM-labeled Col11a1 NTD-derived peptide 3 to fixed monolayers of osteoblastic cells because only biomineralization foci (enriched in bone sialoprotein) and nucleoli (enriched in nucleolin) were visualized. Finally, when added to sections of rat bone, biotinylated peptide 3 was found to bind only to skeletal sites which are known to be enriched in bone sialoprotein, *e.g.*, osteoblastic cells in the basal periosteal layer, forming intramembranous bone, and the tibial secondary spongiosa (12, 13, 33, 35). In contrast, peptide 1 representing the “6a” exonal sequence gave only background binding to bone. Taken together, these results identify for the first time a strong, specific binding interaction between bone sialoprotein and a lysine triplet enriched “6b” alternatively spliced exonal sequence of Col11a1 NTD. We propose that N-biotinylated peptide 3 provides an alternative to antibody-based methods to identify bone sialoprotein protein in western blots and in/on cells in culture and bone tissue *in vivo*. We also speculate that this binding interaction could play a functional role during embryonic new bone formation because other work has shown that type XI collagen can form heterotypic fibrils with type I or type II collagens in which the Col11a1 chain NTD domain and specifically the “6b” sequence is exposed on resultant fibril surfaces at the gap region (38, 39). This newly demonstrated affinity of BSP for the “6b” exonal sequence provides a novel hypothetical mechanism to localize a phosphoprotein mineralization nucleator to collagen fibrillar surfaces of forming bone. However, further work is necessary to confirm this latter hypothesis.

Double labeling studies clearly show that biotinylated peptide 3, a peptide representing a portion of the 6b exon of the Col11a1 NTD containing three lysine triplets, specifically binds to bone sialoprotein within intramembranous new bone. Staining for biotinylated-peptide 3 co-localizes closely with that for monospecific antibone sialoprotein antibodies when both are added to sections of forming intramembranous bone. On day 4 after medullary ablation, a fracture healing model, the intramedullary cavity of tibias is comprised of a rather homogeneous fibrin clot (40). However, at discrete locations, semicircular or spherical thin layers of condensed matrix can be visualized against this background in brightfield. Importantly, both anti-BSP antibodies and biotinylated peptide 3 bind to these condensed matrices and adjacent associated mesenchymal/preosteoblastic/osteoblastic cells. These structures appear to define “mineralized centers” which represent one of the earliest morphological hallmarks of intramembranous bone formation shortly before the first mineral crystals are deposited therein (12, 41). Our results provide biochemical definition for these so-called mineralization centers that has not been previously presented. Specifically, at these sites, we showed here that BSP is contained within thin-walled, closed, extracellular matrix structures with hollow centers where the thickness of the walls are remarkably constant (5–15 microns). Taken together, our histological labeling studies with biotinylated peptide 3 demonstrate that this approach represents a straightforward alternative to antibody-dependent methods to localize bone sialoprotein in western blots, cultured cells, and musculoskeletal tissues.

Because of its extended polyacidic amino acid sequences and its phosphoprotein and sulfoprotein nature, bone sialoprotein has long been viewed as a presumptive nucleator of bone mineralization. BSP can bind many calcium ions (2) and through its strong affinity for hydroxyapatite can modify its crystal growth properties (42). Direct *in vitro* nucleation assays have provided clear evidence of a robust nucleation capacity (3, 43). Also, BSP has been localized to sites of initial mineral crystal deposition termed biomineralization foci in osteoblastic UMR106-01 cells (13), as well in new intramembranous bone formed in the marrow ablation bone fracture model (12). Interestingly, a skeletal phenotype for *IBSP* null mice is evident in embryos and in young mice but not in adults. At birth, *IBSP* null mice are smaller (44). At 3 weeks of age, the growth plate proliferative zone is thinner, and the hypertrophic zone is thicker in null mice (45), although the width of the entire growth plate was not different than wildtype (46). This suggests a regulatory difference in chondrocyte proliferation and apoptosis as well as an alteration in the developmental transition from cartilage to bone (46). At 4 months of age, null mice display thinner cortical bones than WT but exhibit greater trabecular bone volume with a diminished bone formation rate and bone resorption rate. Null mice also exhibit a delay in intramembranous bone formation with a wider cranial suture (45). Thus, while the skeletal phenotype of BSP null mice is complex, it is consistent with an active role for BSP in intramembranous bone formation as well as stimulating chondrocyte proliferation and differentiation of chondrocytes into preosteoblasts (47) during endochondral bone formation. A role for BSP in collagen-mediated nucleation of calcium hydroxyapatite crystals was suggested by an enhancement of BSP nucleating capacity in the presence of type I collagen (48, 49). The binding site was defined as residues 19 to 42 of the Col1a1 chain which contains a conserved sequence with a mix of acidic and basic amino acids (49). However, a collagen binding site has yet to be identified on native type I collagen because BSP has little effect on type I collagen fibrillogenesis *in vitro* (50).

Type V/XI are minor fibril-forming collagens. Owing to their homologous sequences and their ability to interchangeably form heterotypic trimeric collagen structures, type V and XI collagens are considered a single collagen type (22). In Figure 2, type XI collagen is shown as a heterotrimer of Col11a1, Col11a2, and Col11a3 chains. The pro-Col11a1 chain has a large NTD domain which is partially retained after secretion and is subject to alternative splicing. Six prominent alternative splice variants are known to display dramatically different protein sequences. When included, “6b” exon contains a unique motif, *e.g.*, a sequence with four lysine triplets. Also, expression of the “6b” containing NTD isoform is the most tissue-restricted of all possible alternatively spliced isoforms of the type Col11a1 chain (51). Developmentally, it is first localized in embryonic long bones where mineralized tissue initially forms and is later restricted to perichondral regions of cartilage that will subsequently be converted into bone (38, 52).

**Figure 9:**
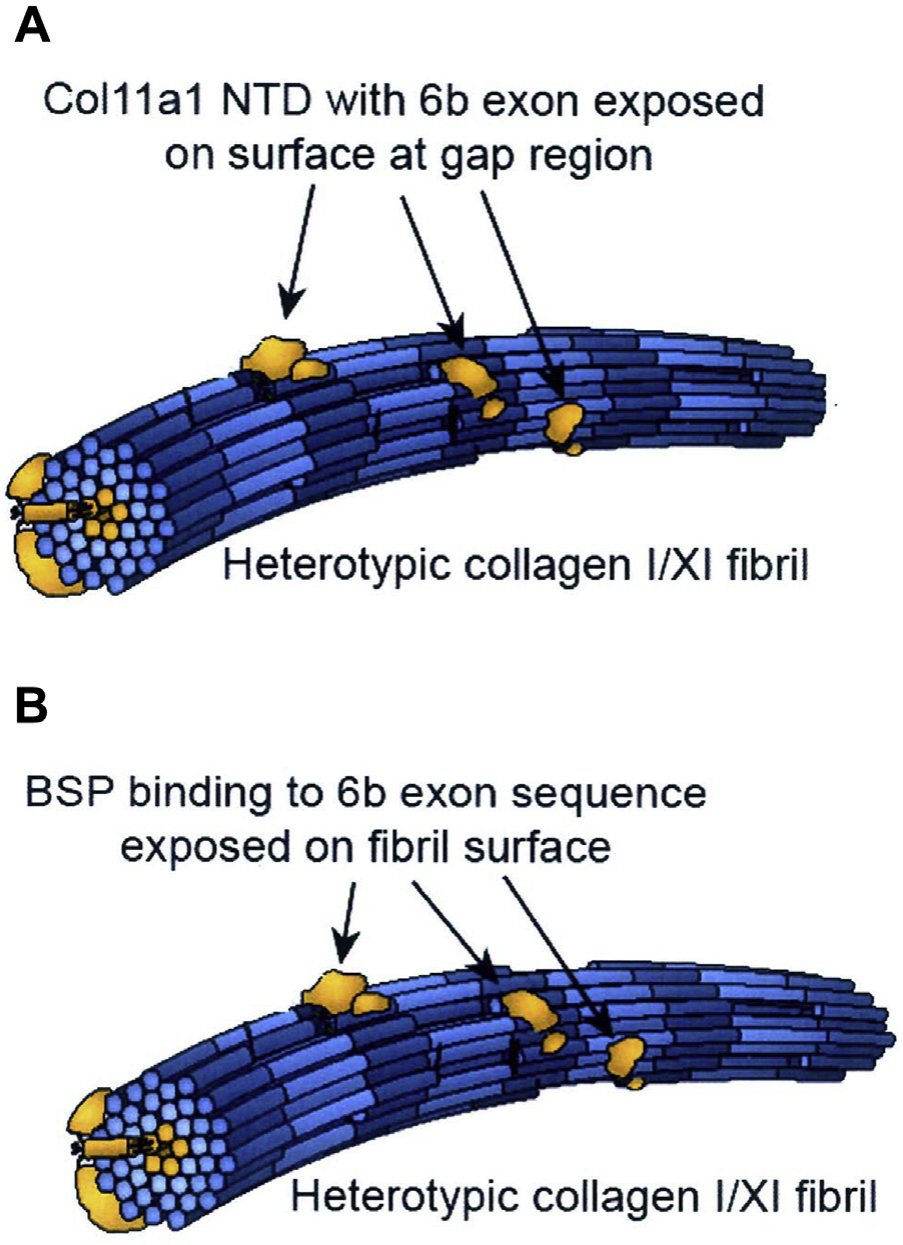
**Hypothetical model: BSP binding to Col11a1 NTD domain containing 6b exonal sequence exposed on surface of heterotypic type I/XI collagen fibrils.***A*, structural model adapted from that published by Eyre *et al.* (39). Type XI collagen (*gold coloring*) is localized at the core of small heterotypic type I collagen fibrils. The Col11a1 chain NTD domain with “6b” sequence (*gold coloring*) projects outward from the surface of the hybrid type I collagen fibrils (depicted as *long rods* with alternating *light* and *dark blue coloring*). *B*, based on evidence presented here that bone sialoprotein specifically recognizes the Col11a1 NTD “6b” exonal sequence, we hypothesize that this binding site will localize bone sialoprotein to the surface of type I collagen fibrils at sites of developmental bone formation. BSP, bone sialoprotein; NTD, N-terminal domain.

Studies of fibrillogenesis have shown that type V/XI collagen can facilitate the nucleation and assembly of type I and type II collagen fibrils (23). In this process, type XI collagen and type I or II collagen initially co-assemble to form heterotypic fibrils. Importantly, the NTD domain of Col11a1 is at least partially retained after secretion and proteolytic removal of N-propeptides from the Col11a2 and a3 chains (53, 54). Exposed on the surface of resultant type I and II collagen fibrils at the gap region (39), the NTD domain of the Col11a1 chain is theoretically available to interact with other collagen fibrils as well as bone noncollagenous proteins and proteoglycans (38, 53) (Fig. 9).

*Cho/cho* mice, which are missing the Col11a1 chain, develop chondrodysplasia in cartilage with sparse, abnormally thick fibrils, despite production of type II collagen normally (55). Histologically, *cho/cho* mice also demonstrate a dramatic increase in trabecular bone within the metaphyseal region at day E18 during development (56, 57). It is noteworthy that a general feature of Col11a1 chain NTD “6b” exon expression during development is its localization as a tight thin layer immediately below the perichondrium. Developmentally, both cartilage and the perichondrium follow programs of coordinated differentiation that originates at the midpoint of the diaphysis, spreading progressively distally from there in both directions. Morris *et al.* (38) have speculated that subsequent events in cartilage development (vascular invasion, endochondral ossification, etc.) may depend upon crosstalk of signals between the perichondrium and associated chondrocytes. Interestingly, “6b” and “6a” expressing Col11a1 NTD fragments (15–60 kDa) have been shown to be present within developing cartilaginous tissues (52); however, only “6b” expressing peptides were shown to negatively influence the expression of differentiation marker alkaline phosphatase by osteoblastic cells (58, 59). When combined with an activating effect of “6b” morpholino-based inhibition of alkaline phosphatase expression (59), these results suggest that proteolytic production of “6b” expressing peptide fragments (52) may play a role in feed forward or feedback regulation of initial steps in perichondrial bone formation.

Peptide binding to western blots containing osteoblastic cell extracts revealed that Col11a1 NTD “6b” exon derived peptide 3 specifically bound to a 110 kDa nucleolin band. This assignment was confirmed by mass spectroscopic peptide mapping. Interestingly, this finding is also supported by prior proteomic studies using affinity chromatography with Col11a1 full length NTD fragments to isolate binding partners from cartilage extracts (60). Nucleolin is located at several sites within cells, *e.g.*, in the nucleolus, in the cytoplasm, and on the cell surface where it forms complexes with growth factors and viral particles (61). Nucleolin displayed a similar specificity toward Col11a1 NTD-derived peptides #1 to 5 as did bone sialoprotein wherein both proteins clearly preferred peptide 3 which contained three triplet lysine sequences. Interestingly, binding of nucleolin to various ligands at the cell surface including HIV particles can be blocked by pseudopeptide HB-19 (62). The structure of HB-19 is composed of a core peptide [KKKGPLEKAhx_CONH2_] which is coupled *via* five of its free amino groups to the pseudopeptide Ks*φ*(CH_2_N)PR. In this way, Col11a1 NTD “6b” exon derived peptide 3 appears analogous to HB-19 in that both peptides contain either multiple consecutive lysine residues or lysines in close proximity three dimensionally. Taken together, this prior work validates our current findings with peptide 3 although it does not immediately suggest a physiological role for nucleolin binding to the Col11a1 NTD “6b” exon sequence. Rather in view of the secreted nature of type XI collagen, we presume that under normal conditions nucleolin and Col11a1 expressed protein would not co-exist within the same intracellular location, *e.g.*, cytosol or nucleolus. However, we can envision a situation where cell surface nucleolin could hypothetically interact with the extracellular Col11a1 NTD “6b” exon sequence.

Data presented here reveal a specific binding interaction between bone sialoprotein and bone restricted alternatively spliced 6b exon of Col11a1 NTD. The individual properties of these two macromolecules and their co-localization to sites of new bone formation provide the basis for us to speculate that complexes of type V/XI collagen, and bone sialoprotein plays a functional role during perichondral and intramembranous *de novo* bone mineralization. In contrast to lamellar bone growth and remodeling which deposits new mineralized matrix onto a mineralized bone/cartilage surface, perichondral bone and membranous bone formation are developmental processes where new bone is formed *de novo* in the absence of a mineralized substrate. Much of our current information derives from a detailed analysis by Bianco *et al.* (33) who have termed the process “vis-à-vis” bone formation. In particular, periochondrial bone formation starts with secretion of a matrix between cells organized into rows where chondrocytes with osteoblastic traits comprise one of the two rows of cells. Importantly, *IBSP* expression coincides both temporally and morphologically with the appearance of the first spherical mineralization centers at the interface between these cartilage and osteogenic matrices (33). Since bone sialoprotein can function as a mineralization nucleator, its presence at this transition between cartilage and bone could hypothetically ensure a seamless mineral phase bridging this transition zone. Interestingly, these authors have also shown that *IBSP* expression within these opposing cell populations peaks at the time of mineralization of the interfacial region and then drops off dramatically (33). Although the mechanism for localization of BSP at this site is unknown, we hypothesize the ability of type V/XI collagen to initiate formation of both type I and type II collagen fibrils and to form heterotypic fibrillar assemblies (23, 26, 27) is key because these hybrid fibrils could bridge this interface between cartilage and new bone. Specifically, the lysine triplet enriched “6b” exonal NTD sequence has been shown to be exposed at gap regions and on the surface of type I (and type II) collagen fibrils (Fig. 9) (29, 39). The newly demonstrated strong affinity of BSP for the “6b” exonal sequence provides a novel hypothetical mechanism to localize a phosphoprotein mineralization nucleator to collagen fibrillar surfaces of forming bone. Further support for this hypothesis comes from Oxford *et al.* (38) who clearly showed that type XI collagen NTD “6b”-expressing protein is restricted to a thin osteogenic layer immediately underlying the perichondrial layer in day E16-E18 embryonic diaphyseal bone—the same region shown by Riminucci *et al.* (33) to express BSP and initiate mineralization. However, further work is necessary to confirm this latter hypothesis.

In summary, the data presented here focus attention on the unique structure and potential functionality of the lysine triplet enriched Col11a1 NTD “6b” exonal sequence. Our findings demonstrate that Col11a1 NTD derived peptide 3 forms complexes with bone sialoprotein that are resistant to high salt. In this way, when coupled with fluorescent detection, biotinylated peptide 3 represents the first nonantibody-based method to specifically identify bone sialoprotein protein on western blots and in/on cells in culture and in skeletal tissues. In addition to its ability to form complexes with bone sialoprotein, several other properties of the lysine triplet enriched Col11a1 NTD “6b” exonal sequence suggest this complex could play a role in initiation of perichondral bone formation. 1) It is localized on the surface of type I and type II collagen fibrils (39) and 2) it’s expression is restricted to the osteogenic layer underlying the perichondrium of developing bone(29). Consistent with a shared role for BSP and Col11a1 complexes in embryonic bone development, *IBSP* null and *COL11a1* null mice both display a similar bone phenotype: an unusually large diaphyseal trabecular bone volume and a very thin cortical bony collar (44, 56, 57). In contrast, expression of the alternative spliced Col11a1 chain NTD “6a” exonal sequence lacks this distinct localization within developing bone. While it remains to be established *in vivo* what the direct functional consequences of this binding interaction are, it is reasonable based on the recent example of asfotase to propose that its existence could have an immediate impact on efforts to treat musculoskeletal disease. Asfotase alpha is a recombinant, catalytically active, duplex form of alkaline phosphatase which contains a polyaspartic acid extension at its two Ctermini (63). The poly-Asp_10_ acid extension facilitates binding of the recombinant protein to exposed hydroxyapatite surfaces on bone tissues. In this way, injected asfotase localizes to bone and provides an effective therapeutic solution to the hypophosphatasia which is the primary cause of hypomineralization in perinatal and infantile hypophosphatasia (64, 65). We believe that the specific binding interaction identified here between the lysine triplet motif of Col11a1 and bone sialoprotein could be more effective than poly-Asp_10_ in targeting materials to bone. Specifically, in contrast to poly-Asp_10_, peptide 3 was shown here to localize to both osteoblastic cells as well as to extracellular matrix sites in forming new bone expressing bone sialoprotein. In this way, peptide 3 or an optimized analog could be used to target agents (toxins, drugs) to inhibit the growth of or kill bone tumor (osteosarcoma) cells. In addition, because localization of peptide 3 or an optimized analog to bone does not rely upon binding to calcium hydroxyapatite, this sequence could be used to target inhibitory agents to immature, poorly mineralized bone prevalent in diseases such as Paget’s. Finally, because bone sialoprotein represents a differentiation marker for osteoblastic cells, we believe peptide 3 or an optimized analog could be used to target growth factors to increase the growth of bone in older individuals who express fewer osteogenic stem cells.

## Experimental procedures

### Materials

FAM-N-terminally labeled peptides and N-terminally biotinylated peptides were synthesized by Invitrogen Inc. UMR106-01 osteoblastic cells were a gift from Dr Ron J. Midura, Cleveland Clinic and Fdn. Antibodies against bone sialoprotein (LF-83) were a gift from Dr Larry W. Fisher, NIH-NIDCR. Anti-nucleolin antibodies were obtained from Abcam, Inc. Antibodies against the N-terminal domain of type XI collagen A1 chain (anti-6b exon epitope, anti-8 exon epitope, anti-Npp epitope) were supplied by Dr Julia Oxford. Digoxygenin-labeled Maackia amurensis agglutinin lectin and horseradish peroxidase conjugated goat anti-digoxygenin antibodies were purchased from Sigma Chemical Co.

Supplies, peptides, and reagents were provided as follows: AEBSF [(4-(2-aminoethyl)-benzenesulfonylfluoride HCl)] (EMD Biosciences Inc); growth media (Eagle’s minimal essential media supplemented with Earle’s salts, 1% nonessential amino acids [Sigma-Aldrich]), 10 mM Hepes (pH 7.2), and 10% fetal bovine serum (Hyclone); growth medium containing 0.5% bovine serum albumin (Sigma-Aldrich); mineralization media (growth medium containing either 0.1% bovine serum albumin or 10% fetal bovine serum and 7 mM BGP); dec-RRLL-chloromethylketone (Bachem); Alizarin Red S dye (ICN Biomedicals Inc); 4 to 20% linear gradient gels (ISC BioExpress); PVDF membranes (Millipore Corp); Amersham ECL Plus western blotting detection System (GE Healthcare); SuperSignal West Dura extended duration substrate (ThermoFisher Scientific); calcium reagents I & II (Pointe Scientific Inc).

### Methods

#### Isolation of bone matrix proteins

Rat bone sialoprotein and osteopontin were isolated from rat calvarial bone as previously described (2).

#### Growth and mineralization of osteoblastic cells in culture and extraction of proteins and SDS PAGE

UMR106-01 osteoblastic cells were cultured using an identical lot of fetal bovine serum and mineralized according to a strict standardized protocol (13, 14, 31). To ensure consistency, passage number was restricted to a value previously shown to maintain a series of phenotypic characteristics, *e.g.*, expression of BSP and bone acidic glycoprotein-75, deposition of mineral crystals within spherical biomineralization foci within an 88 h time period, and quantitative deposition of hydroxyapatite crystals within an expected range. When necessary, frozen aliquots of cells were thawed from defined stocks and grown up following a set protocol. Cells were allowed to acclimate through three to four passages after thawing and only used for experiments after phenotypic characteristics were reconfirmed.

In some cultures, mineralization was prevented by treatment with 0.1 mM AEBSF or 40 μM dec-RRLL-chloromethylketone or by withholding β-glycerolphosphate. UMR106-01 cultures were processed using a two-step protein extraction procedure (15). After the media was removed by aspiration and stored, the cell layer was incubated with 0.05 M EDTA for 2 h at 4 °C to remove materials which appear to be largely associated with biomineralization foci, sites of initial mineralization. The EDTA extract was immediately boiled for 5 to 10 min to inactivate proteases and was then dialyzed against 5% acetic acid at 4 °C before lyophilization to dryness. EDTA extracts were rehydrated with SDS sample buffer and 20 mM DTT and were then subjected to SDS-PAGE on 4 to 20% linear gradient gels (66). Subsequently, the remaining cell layer was extracted with 8 M urea and (3-((3-cholamidopropyl) dimethylammonio)-1-propanesulfonate) (CHAPS) detergent in buffer. Urea extracts were clarified by centrifugation for 1 h at 105,000*g*, and then, the supernatant fractions were processed directly for gel electrophoresis, e g., heating and reduction. Coomassie blue prestained globular protein molecular weight standards (BioRad, Inc) were co-electrophoresed with unknowns for estimation of protein mass. After gel electrophoresis, gels were either processed for Western blotting, incubated with Col11a1 NTD-derived peptides, stained for protein with Coomassie Brilliant blue dye or stains All dye, submitted to N-terminal microEdman protein sequencing, or subjected to mass spectroscopic peptide mapping.

#### Direct fluorescent detection of peptide binding to electroblotted protein blots

Purified rat calvarial bone BSP and osteopontin, along with commercially purified bovine serum albumin, were used in binding studies with Col11a1 NTD peptides. Alternatively, other experiments included cell layer extracts (15) which were electrophoresed on 4 to 20% linear gradient gels. Electroblotting transfer was onto PVDF membrane for 2 h at 100 V. The transfer buffer used was a 10 mM N-cyclohexyl-3-aminopropanesulfonic acid buffer (pH 11.0) containing 10% methanol (15). Blots were washed in Tris-buffered saline containing Tween-20 (TBST), blocked in 5% fat-free milk powder/TBST for 1 h, rinsed with 1× Tris-buffered saline twice, and then blocked with streptavidin and with biotin for 10 min each (Biotin blocking solutions, Vector Labs, Inc). After rinsing the blots with 1× Tris-buffered saline twice, blots were then incubated overnight at 4 °C with blocking solution containing 5 μg/ml of N-terminal biotin-labeled Col11a1 chain NTD peptides #1-#5. After washing multiple times, blots were then incubated for 2 h in the dark with rhodamine red–conjugated streptavidin (3 μg/ml) in blocking solution. Each blot was routinely incubated in 10 ml of streptavidin solution. After multiple washing steps with TBST, blots were imaged in a Fuji LAS 4000 ImageQuant using blue LED illumination with a Y515 filter.

#### Western blotting using chemiluminescence detection

For Western blots, initial processing of PVDF membranes followed a similar protocol as that described above for detection of bound fluorescent peptides to PVDF protein blots. However, after blocking with casein/TBST (ThermoFisher, Inc) for 4 h, PVDF membranes were incubated overnight at 4 °C with primary antibody diluted in 1× casein/TBST (ThermoFisher, Inc). Primary antibody was removed with multiple washings, and blots were then incubated with horseradish peroxidase–conjugated secondary antibody diluted in 1× casein/TBST for 2 h at 4 °C. Blots were finally washed multiple times to remove free secondary antibody and then incubated with ThermoFisher SuperSignal West Dura extended duration substrate and then imaged in a Fuji ImageQuant LAS 4000 Imager.

#### Micro-Edman N-terminal protein sequencing

Proteins were purified by SDS PAGE as described above and then submitted as gels slices to the Macromolecular Structure, Synthesis, and Sequencing Facility, Department of Biochemistry, Michigan State University, East Lansing, MI, on a fee for service basis.

#### Peptide mapping on gel slices using mass spectrometry (Eyre lab)

Protein bands were cut from SDS PAGE gels and subjected to in-gel trypsin digestion (39, 67). Electrospray MS was performed on the tryptic peptides using an LCQ Deca XP ion-trap mass spectrometer equipped with in-line liquid chromatography (Thermo Finnigan LLC) using a C8 capillary column (300 μm × 150 mm; Grace Vydac 208MS5.315) eluted at 4.5 μl/min. Seaquest search software (Thermo Finnigan LLC) was used for peptide identification using the NCBI protein database.

#### LC-MS/MS identification of peptides/proteins (Keightley lab)

Acetone precipitated BMF samples isolated by laser capture microscopy were reduced and alkylated, sequentially, and split into different digests: trypsin or dual digestion with trypsin plus Glu-C. The digests were purified by SPE (ZipTip, Millipore), and extracted peptides were analyzed by capillary LC-tandem MS on a 10 cm capillary column (50 μm ID) packed with Phenomenex Jupiter C18 reversed phase matrix, resolved with a linear gradient of acetonitrile with a flow rate of 250 nl/min. The mass spectrometer (Thermo Finnigan LTQ MS system) was operated in data-dependent mode, with eight dependent scans per survey scan. LTQ.RAW data files were subjected to peak picking with Proteo Wizard 3.0 using generic defaults, with output passed to Mascot *via* Mascot Daemon. Protein identifications were made using Mascot Server 2.6 (Matrix Science) searching against a custom database containing common contaminants (246 proteins), and nine proteins previously found in BMF, with decoy db (reverse) enabled. Mass tolerances for database searches were 1.9 Da for peptide mass and 1.0 Da for fragment peaks. Up to two missed cleavages were allowed. Mascot scores (protein) and individual PSM Ions Scores are reported, with expect values (all meeting at least *p* < 0.05). Individual peptide spectral matches were inspected manually for adherence to expected fragmentation, and neutral loss peaks for phosphorylated peptides (which were also assigned by Mascot).

#### N-biotinylated collagen peptide binding to UMR106-01 cell cultures

Col11a1 NTD derived peptides #1 to 5 were first screened for binding to UMR106-01 cells grown on glass Fisher Super plus microscope slides. Cells were plated onto fibronectin coated slides and grown as described previously (14) [Please also note general comments above about the routine procedures taken to ensure consistency in the growth and mineralization of UMR106-01 cells in culture.]. Cultures were stopped at 70 h by fixation in 70% ethanol for 2 h at 4 °C and then rehydrated in phosphate buffered saline. Cells were removed from three lines dividing each slide into three sections; liquid wax was applied so as to separate each cell section. Endogenous biotinylated sites were blocked by incubation with streptavidin blocking solution for 15 min (Vector Labs), rinsed with 0.05 Hepes buffer (pH 7.5) containing 0.15 M NaCl, incubated with biotin blocking solution (Vector Labs, Inc) for 15 min and then rinsed again with 0.05 M Hepes buffer (pH 7.5) containing 0.15 M NaCl. Biotinylated peptides #1 to #5 (0.5 μg/ml) in phosphate buffered saline were then added to blocked slides in a humidified chamber for 4 h at room temperature. Excess peptide was removed, and cells were washed thoroughly before addition of 3 μg/ml rhodamine-conjugated streptavidin (Jackson Immunoresearch Inc) in Hepes buffer. After 30 min, slides were thoroughly washed with Hepes buffer (pH 7.5) in Coplin jars, slides were drained dry, cover slipped with mounting media, and then observed with a fluorescence microscope. Peptide #3 was the only peptide which showed detectable binding to cultures.

Peptide binding to UMR106-01 cells was quantitated using monolayers grown in 12-well (3.5 cm^2^ surface area) culture plates under standard conditions as described previously (13, 14, 31). Each condition was carried out in quadruplicate (*e.g.*, time after plating, with or without beta-glycerolphosphate). Cultures were stopped at 64 h, 70 h, and 77 h (prior to deposition of mineral crystals) by removal of the media fraction and mild fixation in 70% cold ethanol. (In parallel, separate cultures were processed at 88 h for calcium analysis to confirm their mineralization capacity.) The ethanol was then removed, and dishes were rinsed carefully with 0.05 M Hepes buffer (pH 7.5) containing 0.15 M NaCl. Cell layers were then blocked sequentially for 15 min each with streptavidin blocking solution and then with biotin blocking solution (Vector Labs, Inc). Blocked wells were incubated overnight with gentle rocking at 4 °C with 10 μg/ml of biotinylated peptides #1 to #5 in Hepes buffer. Upon completion, wells were rinsed 3× with Hepes buffer, and cell layers were then incubated for 4 h with rhodamine-conjugated streptavidin (Jackson Immunoresearch) (3 μg/ml) in Hepes buffer. Wells were then rinsed 3× with Hepes buffer, wells drained, and cells dislodged and scraped into 0.05 M Hepes buffer (pH 7.5) containing 0.15 M NaCl and 0.5% Tween 20. Cell suspensions were transferred to white fluorescent 96-well microtiter plates, and fluorescence readings were then obtained with a plate reader. Replicate readings were averaged, and statistical analyses performed as described below.

#### Ion-exchange chromatography on BioCad system

UMR106-01 cells were plated in four 150 mm T-flasks. At 64 h later, the media was changed for serum-free mineralization media containing 1 mg/ml bovine serum albumin and 7 mM BGP (14). At 88 h, the media was removed, and then, the cell layer from each flask was extracted with 50 mM EDTA as described above, pooled, heated to 95 °C, and then inhibitors added to a final concentration of 0.1 mM AEBSF, 10 μg/ml soybean trypsin inhibitor, and 0.5 μg/ml E-64. The pooled extract was dialyzed at 4 °C against 0.05 M sodium acetate buffer (pH 5.2) containing 8 M urea and 0.02% sodium azide (column buffer). EDTA extract was applied to a 10 ml column of strong anion exchange resin (POROS 20 HQ) and washed through with column buffer containing 0.1% CHAPS. The column effluent was continuously monitored at 214 and 280 nm as well as *via* conductivity. A linear gradient of from 0 to 0.6 M NaCl in column buffer containing 0.1% CHAPS was then applied. Finally, a step gradient column buffer containing 2 M NaCl and 0.1% CHAPS was used to elute tightly bound proteins. Aliquots of each fraction were removed for screening by dot-blot analysis, SDS PAGE, and Western blotting.

Five microliters of each fraction were applied to activated PVDF membranes using a Bio-Rad dot blot apparatus (Bio-Rad, Inc). Membranes were processed for chemiluminescence detection as described for Western blotting above with monospecific antibodies for bone sialoprotein and for Col11a1 NTD “6b” epitope. Blots were photographed using a Fuji ImageQuant LAS4000 charge-coupled device camera.

#### Immunostaining of bone sialoprotein in musculoskeletal tissues

Young male rats were subjected to tibial marrow ablation surgery according to a UMKC ALACC committee approved protocol #1103 (JPG) as described previously (40). On day 4 after ablation, animals were sacrificed and tibias were harvested for histological analysis. Following fixation and decalcification, tibias were embedded in paraffin and then cut longitudinally into 5 micron sections. Sections were deparaffinized in xylenes, rehydrated in a graded alcohol series, blocked with 5 mg/ml normal goat IgG for 2 h, and then treated consecutively with streptavidin/biotin blocking solutions (Vector Labs, Inc). Immunostaining was carried out overnight with rabbit anti-bone sialoprotein antiserum (LF-83) (generous gift of Dr Larry Fisher, NIDCR, NIH). Bound antibody was detected with goat anti-rabbit IgG (H and L) antibody conjugated with Alexa 488. Concurrently, sections were also treated with (50 μg/ml) N-biotinylated peptide 3 or control peptide 1 which was detected with Alexa 594-conjugated streptavidin. Alternatively, in some cases, primary antibody was omitted from control sections. Sections were imaged with a Keyence fluorescence microscope and/or a Leica TCS SP5 II Laser Scanning confocal microscope in resonant scanner mode (Leica Microsystems) with line averaging set to 96. Confocal images and z-stacks were captured using a Leica 20× plan apo objective (N.A. 0.7) and a Leica 40× immersion oil objective (N.A. 1.25). For imaging Alexa 488, 488 nm laser excitation was used with an emission collection window of 495 to 560 nm acquired together with a brightfield image. For imaging Alexa 594, laser excitation was 594 nm with a collection window of 610 to 695. For presentation, images were uniformly optimized for size, brightness, and contrast with Photoshop 2020.

### Statistical analysis

Means, standard deviations, median, interquartile ranges (25th to the 75th percentile), minimums, and maximums were calculated for the amount of bound fluorescent peptide for each peptide type and time period combination. Associations between peptide type and the outcome variable within each time period were evaluated using either a 1-way ANOVA or Welch’s ANOVA (if equal variance assumption was violated) with Dunnett’s posthoc tests to compare all peptide types to the buffer alone group. The same statistical tests were used to determine associations between time periods and the amount of bound fluorescent peptide within each peptide type (Dunnett’s posthoc test compared all time periods to the 64 h time point period). All analyses were performed in SPSS v26.0. Depending upon the particular comparison, the significance level is listed as either *p* < 0.05 or *p* < 0.005.

All other experiments were qualitative, and experimental results presented are representative of at least three individual replicates.

## Data availability

Mass spectral data files and searches referenced in Table 1 and in Figure 5*B* are available on request from the corresponding author. The LTQ data from UMKC have also been submitted to the ProteomeXchange database [submission reference #1.20210108-73706]. Data supporting Edman protein sequencing assignments referenced in Figure 5*B* are available on request from the corresponding author. Experimental results referred to as “not shown” or “unpublished results” are available from the corresponding author. All other data are presented within the manuscript.

## Conflict of interest

The authors declare that they have no conflicts of interest with the contents of this article.
